# Natural-Language-Driven Multimodal Representation Learning for Audio-Visual Scene-Aware Dialog System

**DOI:** 10.3390/s23187875

**Published:** 2023-09-14

**Authors:** Yoonseok Heo, Sangwoo Kang, Jungyun Seo

**Affiliations:** 1 Department of Computer Science and Engineering, Sogang University, Seoul 04107, Republic of Korea; ysheo419@sogang.ac.kr (Y.H.); seojy@sogang.ac.kr (J.S.); 2 School of Computing, Gachon University, Seongnam 13120, Republic of Korea

**Keywords:** multimodal deep learning, audio-visual scene-aware dialog system, event keyword driven multimodal representation learning

## Abstract

With the development of multimedia systems in wireless environments, the rising need for artificial intelligence is to design a system that can properly communicate with humans with a comprehensive understanding of various types of information in a human-like manner. Therefore, this paper addresses an audio-visual scene-aware dialog system that can communicate with users about audio-visual scenes. It is essential to understand not only visual and textual information but also audio information in a comprehensive way. Despite the substantial progress in multimodal representation learning with language and visual modalities, there are still two caveats: ineffective use of auditory information and the lack of interpretability of the deep learning systems’ reasoning. To address these issues, we propose a novel audio-visual scene-aware dialog system that utilizes a set of explicit information from each modality as a form of natural language, which can be fused into a language model in a natural way. It leverages a transformer-based decoder to generate a coherent and correct response based on multimodal knowledge in a multitask learning setting. In addition, we also address the way of interpreting the model with a response-driven temporal moment localization method to verify how the system generates the response. The system itself provides the user with the evidence referred to in the system response process as a form of the timestamp of the scene. We show the superiority of the proposed model in all quantitative and qualitative measurements compared to the baseline. In particular, the proposed model achieved robust performance even in environments using all three modalities, including audio. We also conducted extensive experiments to investigate the proposed model. In addition, we obtained state-of-the-art performance in the system response reasoning task.

## 1. Introduction

With the development of multimedia systems in wireless environments, multimodal interactive systems aim to communicate with humans via speech, facial expressions, gestures, and other modalities, resulting in further complexity in human–computer interaction [1]. However, all these technologies are based on the convergence of multiple types of intelligence, including language, vision, audio, and so on, which is still a very challenging task in the whole research domain. Such multimodality started with the convergence of visual and language intelligence, such as visual question answering [2,3,4], and image captioning [4,5,6,7,8]. With the increasing success achieved by using large numbers of image–text pairs in the transfer learning paradigm, interests have recently turned to the video domain. Video captioning [9,10,11,12,13,14,15,16] is the task of describing a visual scene from a given video in a natural language. It is much more challenging than image captioning, which only addresses a static piece of information, in that it requires a comprehensive understanding of multiple frames over the entire video. In fact, from a visual perspective, the key is that the model should have the ability to understand not only the static information but also the dynamics presented over multiple frames. Also, from a linguistic point of view, it should be able to generate coherent descriptions.

From the beginning of the work proposed in [17], research has extended to integrate auditory information. The work [17] first introduced an audio-visual scene-aware dialog task involving interaction with humans via a comprehensive understanding based on multiform information [18,19,20]. The premise of this task is the understanding of accurate visual information, and of the various methodologies developed to generate accurate system utterances based on multimodal data comprising visual and language information. In particular, recent studies [21,22] have mainly focused on a method of leveraging transformer-based language models to integrate individual information obtained from modality-specific feature extractors. Pasunuru and Bansal [23] exploited a dual-attention mechanism to fuse information from multiple modalities. Li et al. [22] proposed a transformer-based multimodal dialogue generation framework that can integrate all the modality information in a language model.

Despite the initial success of multimodal integration of the three modalities, there have been problems in bridging the gap to reach commercialization. First of all, a standardized method for effectively using audio information has not yet been proposed. Schwartz et al. [24] have proposed a co-attention-based multimodal fusion algorithm. It has shown impressive performance only with vision and text knowledge. This tendency has led to the maintenance of a transformer-based approach [22]. More importantly, these works were heavily dependent on summaries that contained the overall information of the scene as a natural language. The finding shows that the performance drop occurs without summaries in inference time.

Therefore, we propose a novel audio-visual scene-aware dialog system that utilizes a set of explicit information from each modality as a form of natural language, which can be fused into a language model. Then, the model is able to generate the appropriate answer to a given query. We also propose a multitask learning method using the summary generation problem as an auxiliary task to better understand multimodal information and generate a more robust response. To the best of our knowledge, this approach has not been explored yet. However, it can address the existing limitations in a robust way. In addition, we propose a response-driven temporal moment localization method to strengthen the interpretability of the system response generation process. The system itself provides the user with the evidence referred to in the system response process as a form of the timestamp of the scene. The performance showed robust generation capabilities compared with the baseline model. We also conducted extensive experiments to investigate the model. In addition, we obtained state-of-the-art performance in the system response reasoning task. The contributions of this paper can be summarized as follows:

- We introduce a novel audio-visual scene-aware dialog system with natural-language-driven multimodal representation learning through which the system can infer all information by sequentially encoding the keywords obtained from each modality into the transformer-based language model;
- We also propose a response-driven temporal moment localization method in which the system itself provides the user with the segment of the video that the system referred to for response generation;
- In addition to the ability to generate responses with improved quality, the proposed model showed robust performance even in an environment using all three modalities of information, including audio. With regard to the system response reasoning task, our proposed method achieved state-of-the-art performance.

The remainder of this paper is organized as follows. Section 2 introduces related works. Then, the explanation of the proposed architecture is addressed in Section 3. In Section 4 and Section 5, we describe the experiment and discussion, and then finalize the paper in Section 6.

## 2. Related Works

### 2.1. Video-Grounded Text Generation

Video-grounded text generation is the generation of a text given a video. This task needs to address the convergence between video and text. At the beginning of the study, rule-based approaches [25,26,27,28,29] were proposed to produce sentences using a fixed set of predefined templates, a triple consisting of a subject, verbs, and objects. Notwithstanding their high grammatical accuracy, these have strong limitations in terms of the low-complexity rules for sentence construction and generalization. With the growth of deep learning, encoder–decoder-based architectures have been utilized in various ways. SCN [30] is a semantic concept detection method that obtains the probabilities of concepts appearing in a video from CNN. They incorporated concept-dependent information into LSTM to compose semantic representations. SGLSTM [31] introduced a method for jointly evaluating visual and semantic features using two semantic guiding layers by adopting different levels of semantics as guidance to control the language model to generate sentences.

Unlike image-to-text generation tasks, which handle only static moments, video-grounded text generation tasks should address the means for understanding the dynamics that appear across multiple frames in the video. SemSynAN [32] introduced a method to strengthen the understanding of temporal composition by mapping visual concepts to their corresponding part-of-speech tags in text descriptions. In addition, Chen and Jiang [33] introduced a recurrent region-based attention mechanism and motion-guided information control method to selectively capture temporal relationships. Moreover, with the success of transformer-based architectures in most vision-language tasks, SwinBERT [34] proposed a method of incorporating a transformer-based video feature extractor and transformer-based encoder. It showed a considerably high performance in video captioning tasks. Moreover, MVGPT [35] introduced a large-scale video-to-text model using a pretraining-finetuning strategy. It contains a large-scale video understanding model [36] and transformer-based decoder [37] as its backbone. Because the model capacity is the largest among all the works, it has shown significant results. However, it has a high dependency on resources.

### 2.2. Audio-Visual Scene-Aware Dialog

Most recent studies on multimodal dialogue systems were accompanied by a transformer-based network. Huang et al. [21] proposed a multimodal transformer network that obtained individual information from feature extractors for each modality and combined these using a text-based cross-modal attention mechanism. Li et al. [22] proposed a transformer-based generative framework that integrates all the modalities by encoding features into the system and generates better multimodal-based system responses using multi-task learning methods. Chu et al. [38] described a consecutive multimodal fusion strategy using joint modal attention during conversation. Although these approaches exhibit significant performances, they have two limitations. One is that the overall system performance is considerably dependent on the usage of summaries during the training phase [39]. Second, effective multimodal integration strategies using audio have not been demonstrated sufficiently.

## 3. Proposed Architecture

**Figure 1:**
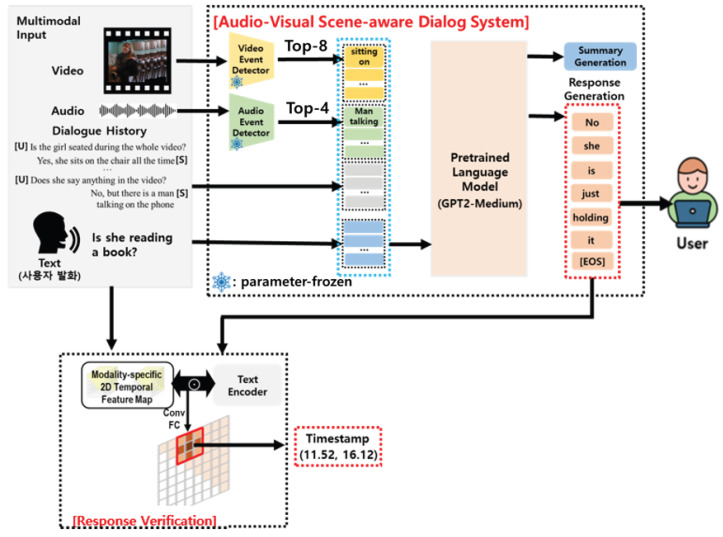
The proposed architecture for audio-visual scene-aware dialog.

In this section, we propose a novel audio-visual scene-aware dialog system with system-generated response verification. As shown in Figure 1, it accepts video, audio, a dialogue history, and the last user query as inputs. It consists of two parts: event-keyword-driven multimodal integration and response generation using a pretrained language model. The first part extracts event keywords from visual and auditory information using modality-specific event extractors. Unlike the previous works [21,22] that employed implicit features from modality-specific encoders, event keywords are explicit information that appears in the scene. Then, this information, including the dialog history, can be combined via iterative encoding into a pretrained language model. In the internal modules in this model, all the information can be integrated into a shared semantic space, which can be fed into the response generation process.

Next, we leverage a pretrained language model to combine all the knowledge and generate an appropriate response by training it in a multi-task learning paradigm. Inspired by the previous works [39] wherein the response generation performance relied significantly on the summary, this model is trained on a new auxiliary task called summary generation. This task generates a summary given a set of event keywords from the visual and audio modalities. Therefore, the language is trained in multi-task learning. This can address the summary dependency issue.

Existing multimodal works [21,22] on the three modalities have two requirements. One is that we require an understanding model for both visual and speech models. The second is that we require a mechanism for fusion. In general, the features obtained from each modality are combined using a self-attention structure. More importantly, these incur significant costs because training accompanied by a large amount of data is essential.

However, a significant advantage of the proposed system is that it can conveniently address this problem. Our proposed model utilizes pre-trained visual and audio event extractors without additional training. In addition, the pre-trained language model enables the model to infer the relationship between the knowledge inherent in the model and the dialogue history related to the event keyword. It also enables meaningful results in the multimodal domain using only fine-tuning for downstream tasks.

Moreover, to verify the system-generated response reasoning, we propose a modality-specific response-driven moment localization network that can identify a temporal segment of a given scene that is semantically similar to a given query and system-generated answer. As described in Figure 1, it provides the user with a basis in the form of timestamps of video fragments referenced by the model in the response generation process. This significantly improves the interpretability of the reasoning process of the system. Each component of the architecture is described in detail in the following subsections.

### 3.1. Event Keyword-Driven Multimodal Integration Using a Language Model

The scene-aware conversations in this study mainly encompass events that appear for the video and audio modalities. More specifically, the events refer to all information such as the activities of objects, background sounds, and object relationships. Therefore, the understanding of multimodal information is directly related to that of the events shown in the scene. Inspired by this fact, we employed pretrained event detectors specialized for each modality to extract various events that occur in the video and regard these to be the information from each modality. Figure 1 shows the example of a woman sitting on a chair with a book and playing with her shoelaces. The video does not contain any specific audio information. In this case, the video event detector predicts event categories such as “holding” and “sitting” with high probability. The top N video event categories correspond to information estimated to have appeared in the scene with a high probability. Therefore, we used these categories as the direct information obtained from the visual modality. This approach has an advantage in terms of multimodal understanding in that it uses more explicit natural language information than that in previous studies that applied feature embedding for each modality. The AVSD data we addressed includes both audio and video. Therefore, in this study, we used a pretrained transformer-based event classification model, which is available to the public, to extract event information for each modality.

#### 3.1.1. Audio Event Detector

In this paper, we adopted audio spectrogram transformer (AST) [40] as the backbone for the audio event detector. AST is the first transformer-based model proposed for audio event classification problems. It constructs an encoder model based on self-attention and feed-forward layers. The input speech is converted into a sequence of 128-dimensional log-mel spectrograms that are used as model inputs. Each spectrogram is divided into patches of a fixed size. The model generates encoded results in units of patches as the output. We regarded the output embedding of “[CLS],” which was the first input token of the model, as the entire embedding information of the audio spectrogram. This embedding was used as the input vector for the audio classification layer. The AVSD data used in this study did not provide audio classification labels for the audio at the scene. This study adopted an open-public AST model. It is a fine-tuned transformer-based encoder with an audio set [41] comprising 527 audio event categories. However, the model did not perform additional training on the model. Furthermore, the M-audio event category results with high probability values for input speech were considered as events detected from speech. In practice, four audio event categories are set as a pivot.

#### 3.1.2. Video Event Detector

In this paper, we adopted a video swin transformer (VST) [42] as a backbone of the video event keyword detector. It exhibits a high performance in video action recognition tasks. This model consists of stacks of swin transformer blocks [43]. A VST utilizes large-sized patches when passing through layers. Moreover, self-attention between multiple patches is performed by altering the locations of the windows in each layer. Next, the model can sufficiently learn the context of the entire image by performing self-attention only between patches within a fixed-size window for each transformer block. We followed the setting in Liu et al. [42] to fully utilize the capacity of the model. We sampled a clip of 32 frames from each full-length video by using a temporal stride of two and a spatial size of 224 × 224. This results in 16 × 56 × 56 input 3D tokens. Similar to an original transformer-based encoder [44] in natural language processing, we considered the embedding of the [CLS] token, i.e., the output embeddings from the VST, as a context for the entire video. Then, it was applied as an input to the linear classification layer for action recognition. In this study, we utilized the smallest VST model fine-tuned on kinetics-400 [45]. This is a large-scale human action dataset for 400 human action categories. The AVSD data used in this study contained no action labels for the scenes. Therefore, this study regarded the 400 predefined human-action categories in kinetics-400 as events that can occur in videos. In addition, the model accepts the N action categories with the highest probabilities in the action recognition layer of the model for the input video as events detected in the video. In practice, eight action categories are set as pivots.

### 3.2. Response Generation

Each modality has a set of event labels in natural language in a given audio-visual scene. This enables the integration of multimodal information by encoding the information from each modality directly into the language model. We sequentially encoded the M audio event labels, N video event labels obtained previously, conversation history, and last user query in a language model. In this study, we utilized it as a language model. GPT2 [37] exhibits good performance in various generative tasks. Specifically, the input configuration of the model is as follows:

(1) F=([AUD],AE,[VID],VE,D)

where AE is a sequence of M audio event labels, VD is a sequence of N video event labels, [AUD] and [VID] refer to the special separator tokens for audio and video event labels, respectively, and D is a sequence of words in the dialog history. In particular, we add two special separator tokens [Q:] and [A:] to the beginning of every question and answer.

**Figure 2:**
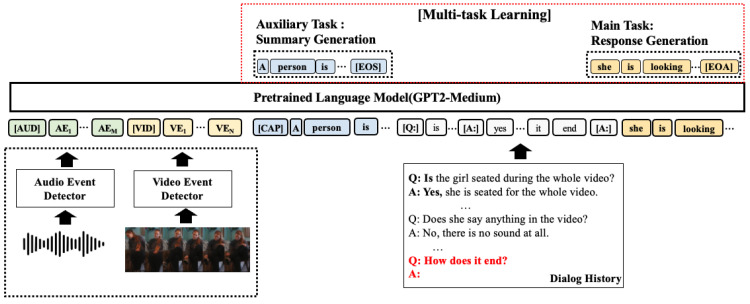
An illustration of response generation based on event keywords, dialog history, and last user query.

Now, we propose multi-task learning for robust response generation using a summary generation task as an auxiliary task. It contributes to a better understanding of event-keyword-based multimodal information. As shown in Figure 2, the summary generation task works to generate a summary of a given audiovisual scene. Specifically, the model sequentially accepts a set of keywords as an input from an audio event detector and a video event detector, and generates an appropriate summary in an autoregressive manner until the end of summary symbols ([EOS]). The response generation task generates a response conditioned by a set of event keywords, a model-generated summary (S), and dialog history (D). The model can be generated autoregressively until the end of the response symbol ([EOA]) is generated.

In a multitask learning setting, the training objective is to optimize the parameters of the language model, θ, by maximizing the weighted sum of the losses for each task:

(2) $$L=\alpha \cdot L_{\mathrm{summary}}+\beta \cdot L_{\mathrm{response}}$$

where α and β are hyper-parameters, and, in this work, values are set as 1. Each loss function is defined as a log-likelihood of generated sequences for each task. More specifically, as for the summary generation task, every token is generated with the highest probability for a given a set of audio event keywords (AE), video event keywords (VE), and previously generated tokens (s), which can be formulated in Equation (3). Similarly, as for the response generation task, each token is chosen with highest probability for given a set of audio event keywords (AE), video event keywords (VE), model-generated summary (S), and dialogue history (D), which can be formulated in Equation (4).

(3) $$L_{\mathrm{summary}}=-\sum_{i=1}^{K}\log P\left(s_{i}|\mathrm{AE},\;\mathrm{VE},s_{<i};\theta \right)$$

(4) $$L_{\mathrm{response}}=-\sum_{j=1}^{L}\log P\left(r_{j}|\mathrm{AE},\;\mathrm{VE},\;S,\;D,r_{<j};\theta \right)$$

where AE, VE, S, D refer to audio event keywords, video event keywords, summary, and dialog history, respectively.

### 3.3. Response-Driven Temporal Moment Localization for System-Generated Response Verification

**Figure 3:**
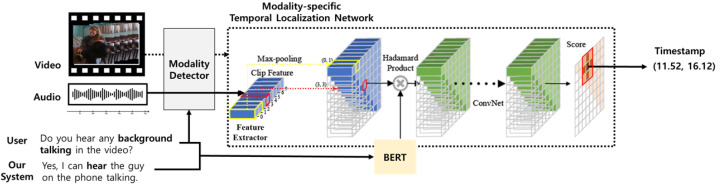
An illustration of a response-driven modality-specific temporal moment localization network. In this case, audio-modality is only used due to the modality detector. This figure is a variant of the one in Zhang et al. [46].

This section describes a response-driven moment localization network that can identify the timestamp of a scene semantically similar to a given query and system-generated answer. A system-generated response should refer to scene segments near an occurrence event related to user queries. More specifically, the system identifies a modality from which an indication of the event can be obtained, analyzes the features of the modality, and uses these to generate an answer to a query. For example, in Figure 3, the user asks whether the woman in the video is talking. In this case, the system requires the voice information of the woman in the video. That is, the system should detect the temporal segment in which the woman is talking using an auditory modality.

Motivated by this observation, this paper proposes a response-driven moment localization network that can identify a temporal segment of a given scene that is semantically similar to a given query and system-generated answer. As shown in Figure 3, the network consists of two parts: a modality detector and modality-specific temporal localization. The first part aims to increase the accuracy of localization by heuristically analyzing whether a user query focuses only on the visual or auditory information in a scene. The second part is used to predict the temporal moment by measuring the similarity between the embedding of each temporal segment from either video or audio, and the embedding of the user query and system response. Each component is described in detail below.

#### 3.3.1. Modality Detection

To enhance the accurate moment localization, we added a query analysis to heuristically identify a specific modality that is highly likely to contain the evidence for the user query. We observed that it was occasionally unnecessary for a system to generate an answer using all the information from different modalities. Rather, it is favorable to use information from a single modality for user queries. As shown in Figure 3, if the system focuses only on information from the auditory modality, it can obtain supporting evidence for a more accurate answer. As a result, we address queries that can be answered with a single modality, and we heuristically determine keywords that frequently appear in queries that can be answered only using information from the auditory modality. Detailed keywords are described in Table 1. In the actual temporal moment localization phase, we use only auditory information for the queries containing the aforementioned keywords. Otherwise, the video stream on the scene is used.

#### 3.3.2. Modality-Specific Temporal Moment Localization Network

We introduce a modality-specific moment localization network that can identify the temporal moment of a scene that is semantically similar to a given user query and the system-generated answers. Specifically, we utilized a variant of the 2D temporal adjacent network (2D-TAN) [46]. The two networks were trained independently according to each visual and auditory modality. An audio-based 2D-TAN is used to identify temporal segments on the audio signal that is semantically similar to the given query and answer when it is determined in the query analysis step. Here, the query can be solved using only audio information. Otherwise, a video-based 2D TAN is adopted to identify the temporal video moments in a video stream.

The audio-based 2D-TAN consists of three steps: natural language encoding, audio signal encoding, and temporal moment prediction. First, we employ BERT to obtain semantic information of the user query and the system-generated answer. In this study, we concatenated these into a sentence and encoded these using BERT. In particular, the output embedding of the [CLS] token of BERT is used as semantic information on the entire sentence. For audio processing each audio signal, we first segmented these into 16 non-overlapping clips. The feature representation for each clip can be obtained by average pooling the audio features of the frames included in the clips extracted from the VGGish model provided by the organizer. Then, similar to [46], the audio signal is encoded in the form of a two-dimensional temporal feature map designed to represent key features appearing across a specific time span by max-pooling features for consecutive clips. Now, the auditory and language information can be combined using the Hadamard product. Moreover, the relevance score between the auditory and natural language sentence can be calculated using a temporal adjacent network with the multiple convolution operations on the combined 2D temporal feature map. Finally, the semantic similarity score between the given query and system-generated utterances and each temporal moment can be obtained in the form of a two-dimensional score matrix. We utilize this instant using the highest value in the score map as the final output.

This process is applied identically to video-based 2D-TAN. The difference is that video features, which are I3D features provided by the organizer, are used. To train two networks independently during the query analysis step, we split samples that can be answered only by the auditory modality from the training data and used these only to train the audio-based 2D-TAN. The other samples were used to train the video-based 2D-TAN. The training process is based on the [46], and we train two networks from scratch using the DSTC10 reasoning data provided by the organizer. Following [46], the training objectives of both models are based on a scaled IoU value as the supervision.

## 4. Experiment

This section addresses the experimental setup and experimental result for the performance of the proposed architecture.

### 4.1. Experimental Setup

#### 4.1.1. Dataset

This work adopts the Audio Visual Scene-aware Dialog (AVSD) dataset [18], provided by the organizers in the tenth dialog system technology challenge (DSTC10, available online: https://github.com/dialogtekgeek/AVSD-DSTC10_Official, accessed on 10 August 2021). During the AVSD data collection, two humans (a questioner and an answerer) conversed regarding the events in a video. Having watched the video, the answerer answered the questions posed by the questioner. The participants were not permitted to watch the video. Rather, they were given three static images (first, middle, and final frames) to establish a basic understanding of the scene. After ten rounds of the question and answering process, the questioner wrote a summary of the video events. This study used a split version of the official validation set for the Charade challenge in half, and used these halves for the validation (1787 videos) and testing sets (1804 videos).

#### 4.1.2. Implementation Details

All the experiments were conducted on a Linux server with Ubuntu 18.04 and 2-GPUs of Nvidia-3090. This work exploited medium-sized GPT2 [37] (355M parameters) as a language model. It was fined-tuned on AVSD datasets with a batch size of 4 for 20 epochs. The training processes were stopped early when there was no progress on the BLEU-4 score of the validation set for the five consecutive epochs. In more detail, we set the learning rate as $2\times 10^{-5}$ with the adamW optimizer and cosine-annealing scheduler. We take 8 video event keywords and 3 audio event keywords as inputs to the language model. We also adopt beam-search as a decoding strategy with a beam size of 3.

As the video event extractor, this work adopted a small-sized video-swin transformer [42] (50 M parameters). For keyword extraction, each video is uniformly sampled in the temporal dimension as 4 clips, and, for each clip, the shorter spatial side is scaled to 224 pixels, which is the same setting as in [42]. As the audio event extractor, this work adopted a small-sized audio-spectrogram transformer [40]. For keyword extraction, each audio is split into separate audio clips with 10 s to match the model’s capacity. The rest of the setting is the same as in [40].

### 4.2. Evaluation Metrics

To compare the quality of the generated responses, we adopted four automatic evaluation metrics widely used in most generation tasks such as BLEU [47], ROUGE [48], METEOR [49], and CIDEr [50]. For the response verification, the automatic evaluation metric was the intersection over union (IoU). It indicates the ratio of overlap between the predicted and human-annotated timestamp. Presumably, a higher score is better. Owing to multiple valid temporal segments for each response, we adopted two types of IoU: IoU-1 and IoU-2. IoU-1 can be measured by an average IoU computed between each ground truth and the predicted timestamps. This provides the highest IoU for the ground truth. IoU-2 can be measured by computing frame-level matching among all the predicted and ground-truth temporal segments for each response.

The result also contained the human evaluation performed by the DSTC10 organizers. They collected human ratings for system responses using a five-point Likert scale. Here, humans rated the system responses given a dialog context as follows: 5: good; 4: good; 3: acceptable; 2: poor; and 1: very poor. They asked the human raters to consider the correctness of the answers as well as the naturalness, informativeness, and appropriateness of the response according to the given context.

### 4.3. Experimental Result

We describe the experiment results in three settings, text + visual, text + visual + audio, text + visual + audio + summary, as shown in Table 2. Based on the BLEU-4 value, the performance in the text + visual + audio + summary setting was the highest.

We first conducted the experiment using only text (question–answer pair) and visual information. As can be seen in Table 2, our model exhibited a high performance in all the metrics compared with the baseline model. It displayed improvements of 0.0477 in BLEU-4 and 0.2234 in CIDEr. In the text + visual + audio setting, the model used audio information as well as the visual information in the video to generate an answer. Our model showed a higher performance than the baseline model in all the metrics in this setting as well. It displayed improvements of 0.0515 in BLEU-4 and 0.2382 in CIDEr. Compared with the text + visual task without audio information, it displayed an improvement of 0.0038 in BLEU-4 and a decrease of 0.0148 in CIDER. Finally, in the text + visual + audio + summary setting, we observed the effectiveness of the multi-task learning method. Specifically, our model showed performance improvements of 0.0517 in BLEU-4 and 0.2211 in CIDER compared with the baseline model. The CIDEr value was marginally lower than that for the text + visual + audio task without summaries. However, the BLEU-4 value was higher.

Meanwhile, MED-CAT showed marginally better results than our proposed model. The evaluation result for BLEU-4 verified that our proposed model was approximately 0.0734 lower. Additionally, in the qualitative evaluation, the proposed model was approximately 0.2 points lower. This was because the capacity of MED-CAT is significantly larger than that of our model. MED-CAT is based on a pretrained model with highly advanced video language understanding tasks called UniVL [52]. Our proposed model also uses a pre-trained event-detection model for video recognition tasks. However, we used only small-sized models because of these limitations in the learning environment. In addition, UniVL is a pre-trained model for videos and language convergence tasks. This is more directly related to the dataset used in our study. Nevertheless, this result is sufficiently significant in that our proposed model showed a performance comparable to that of MED-CAT regardless of the size of the model.

Moreover, as shown in Table 3, for both IoU-1 and IoU-2, performance in the text + visual + audio + summary setting (Table 2) was the highest. In particular, we achieved state-of-the-art performance compared with the reported results in the DSTC10 challenge. More specifically, we conducted the response verification experiment using the response generated by the model with the “T + V + A + S” setting mentioned in Table 2. As shown in Table 3, compared with the baseline models, our model displayed better scores, with large margins of 0.1543 and 0.1645 for IoU-1 and IoU-2, respectively. These comprise the highest IoU-1 and IoU-2 results among all the submissions of DSTC10. More importantly, our result outperformed the MED-CAT model, which has shown better performance on the response generation task.

## 5. Discussion

To analyze the performance of this work from various perspectives, we conducted extensive experiments, such as an investigation of the accuracy of keyword extraction and its effect on response generation.

### 5.1. The Performance of Modality-Specific Event Keyword Extraction

This study utilizes event keywords observed in videos and audios as multimodal information. Therefore, the accurate event keyword prediction directly influenced the system response generation. However, if a discrepancy exists between the video domain of the video event prediction model and the actual video domain, this method has a caveat. Meanwhile, if the video domain is not completely independent, event prediction for new videos should be addressed. In the initial stages of the study, we verified a certain degree of consistency between the video domain used in the training phase and the domains of the videos used for the actual evaluation. A video-domain-independent method of event prediction model will be addressed in future work.

Therefore, it is essential to analyze the accuracy of the event prediction model. The evaluation metrics used in this experiment are average precision@N (P@N), average recall@N (R@N), and average F1 Score (F1). P@N calculates the ratio of actual events observed among the top-K event keywords predicted from the videos. R@N calculates the ratio of actual events observed among all the ground-truth events. F1 measures the harmonic mean of precision and recall. For this evaluation, 50 videos from the evaluation dataset were randomly selected. Since the dataset does not have actual event labels, we have assigned event labels to the videos manually. Similarly, the evaluation for audio was conducted in the same manner.

The prediction of event keywords for videos indicated that the range of the desired predicted keywords increased. Larger numbers of actual answer keywords were included. As shown in Table 4, the F1-Score was highest when N = 10. The precision did not vary significantly. However, the recall increased as the prediction range widened. This phenomenon occurred because as the prediction range expanded, the correct answer keywords were likely to be included. More importantly, this result provides strong evidence for determining the range of video keyword counts to be used in the process of multimodal integration. In contrast to video, audio event prediction showed relatively opposite results. As shown in Table 5, the F1-Score generally increased when the range of desired predicted keywords was narrower. The results indicate that the highest performance in the case was evaluated using three predictions (N = 3). This result was obtained owing to the bias in the perceptible results from the audio. In reality, the correctly predicted cases were mostly limited to a small number of labels such as “man talking” and “background noise”. The majority of the other cases had a lower accuracy. These observations can serve as significant evidence for determining the number of audio events used in the multimodal integration process.

### 5.2. The Effects of the Number of Event Keywords

The previous experiment analyzed the optimal number of events, determined through precision, for the event prediction model to obtain results that include actual events. However, the most important aspect is to analyze how the quality of the generated system responses varies with the variation in event keyword counts. Therefore, this experiment demonstrated the quality of system-generated responses based on the number of predicted event keywords in videos and audio clips. Specifically, the first experiment evaluated the system-generated responses by varying the number of video keywords while maintaining the number of audio keywords constant at three. As shown in Table 6, video event keywords generally exhibit the most robust performance when there are eight keywords. This is because these achieve the highest scores in widely used evaluation metrics for generation research, such as BLEU-4, METEOR, and ROUGE. It can be observed that the response generation capability decreases marginally in the vicinity of eight keywords, except for certain metrics.

As shown in Table 7, audio event keywords generally exhibit the most robust performance when there are four keywords. This is because these achieve the highest scores in the representative evaluation metrics for generation research, such as BLEU-4, ROUGE, and CIDEr. When the number of keywords is at most four, it can be considered that similar response generation results are obtained. However, it is generally observed that as the number of keywords increases, the response generation capability decreases marginally. This observation is supported by qualitative analysis. It indicates that audio requires only structured information such as the protagonist’s voice and the presence of surrounding voices. Therefore, it is conjectured that an unnecessarily large number of audio events may function as noise during the response generation process.

### 5.3. Ablation Study for Response Verification

As shown in Table 8, we conducted an ablation experiment to analyze the effect of the modality detector and the type of modality information. The threshold model was trained using all the modalities, including audio with multi-task learning. When the summaries were not included, the IoU-1 and 2 scores decreased by 0.0096 and 0.0105, respectively. When auditory information was additionally excluded, in the IoU-1, two scores decreased by 0.0013 and 0.0009, respectively. Most importantly, the modality detector had an impact on the performance reductions of 0.0134 (2.60%) and 0.0139 (2.55%), respectively.

## 6. Conclusions

In this paper, we propose a novel audio-visual scene-aware dialog system that can integrate all the information by iteratively encoding the event keywords (which are extracted from modality-specific event extractors) into a pre-trained language model. In addition, inspired by the fact that previous works depended significantly on a scene summary, the proposed system was trained on multi-task learning with summary generation as an auxiliary task. We also propose a response-driven temporal moment localization network by providing evidence that can be used in the response generation process. It can provide users with the reasoning process regarding how the system produces the response by generating the timestamp that the system has utilized before. By integrating this method into the audio-visual scene-aware dialog, the user can interpret the reasoning system process, which is regarded as a “black box.” This contributes to enhancing trustworthy AI systems. In future work, we aim to integrate the proposed system with external symbolic knowledge to develop a more interpretable and robust system.

## Figures and Tables

**Table 1:** A dictionary with 23 audio keywords.

audio, audible, noise, sound, hear anything, can you hear, do you hear, speak, talk, talking, conversation, say anything, saying, dialogue, bark, meow, crying, laughing, singing, cough, sneeze, knock, music, song

**Table 2:** Experimental results for answer generation task on the test set provided by the organizers in the DSTC10-AVSD challenge (T: text; V: visual; A: audio; S: summary).

Models	BLEU-1	BLEU-2	BLEU-3	BLEU-4	METEOR	ROGUE-L	CIDEr	Human Rating
Baseline	0.5716	0.4223	0.3196	0.2469	0.1909	0.4386	0.5657	2.851
Our model								
T + V	0.6409	0.4897	0.3764	0.2946	0.2274	0.5022	0.7891	-
T + V + A	0.6406	0.4885	0.3786	0.2984	0.2251	0.5016	0.8039	-
T + V + A + S	0.6455	0.4889	0.3796	0.2986	0.2253	0.4991	0.7868	3.300
MED-CAT [51]	0.6730	0.5450	0.4480	0.3720	0.2430	0.5300	0.9120	3.569

**Table 3:** Experimental results for temporal localization task on the test set provided by the organizers in the DSTC10-AVSD challenge. The proposed model is trained on multi-task learning with auditory information mentioned by T + V + A + S in Table 2.

Models	IoU-1	IoU-2
baseline	0.3614	0.3798
MED-CAT [51]	0.4850	0.5100
Proposed Model	0.5157	0.5443

**Table 4:** The performance of the video event detector on 50 videos randomly sampled from the validation set.

Top N	Precision@N (P@N)	Recall@N (R@N)	F1-Score (F1)
N = 5	0.333	0.219	0.264
N = 6	0.367	0.291	0.324
N = 7	0.348	0.322	0.334
N = 8	0.358	0.381	0.370
N = 9	0.363	0.439	0.398
N = 10	0.367	0.492	0.420

**Table 5:** The performance of the audio event detector on 50 videos randomly sampled from the validation set.

Top N	Precision@N (P@N)	Recall@N (R@N)	F1-Score (F1)
N = 1	0.30	0.120	0.171
N = 2	0.28	0.223	0.248
N = 3	0.253	0.313	0.280
N = 4	0.22	0.353	0.271
N = 5	0.208	0.409	0.276

**Table 6:** The performance of response generation with respect to the number of video event keywords. The number of audio event keywords is fixed as 3.

# of Keywords (K)	BLEU-1	BLEU-2	BLEU-3	BLEU-4	METEOR	ROUGE-L	CIDEr
K = 3	0.601	0.451	0.347	0.282	0.225	0.499	0.607
K = 5	0.624	0.475	0.366	0.286	0.225	0.502	0.7970
K = 8	0.6455	0.4889	0.3796	0.2986	0.2253	0.503	0.7868
K = 10	0.646	0.489	0.366	0.287	0.231	0.502	0.786

**Table 7:** The performance of response generation with respect to the number of audio event keywords. The number of video event keywords is fixed as 8.

# of Keywords (K)	BLEU-1	BLEU-2	BLEU-3	BLEU-4	METEOR	ROUGE-L	CIDEr
K = 1	0.611	0.4781	0.3511	0.292	0.2254	0.5013	0.717
K = 2	0.657	0.4875	0.3694	0.2911	0.2251	0.502	0.7810
K = 4	0.6455	0.4889	0.3796	0.2986	0.2253	0.503	0.7868
K = 5	0.611	0.4854	0.3610	0.2878	0.219	0.5021	0.694

**Table 8:** Ablation studies for evaluating each component and each modality. The proposed model is trained on multi-task learning with auditory information mentioned by T + V + A + S at Table 2.

Models	IoU-1	IoU-2
Proposed Model	0.5157	0.5443
-S	0.5061	0.5338
-S -A	0.5048	0.5329
-Modality Detector	0.5023	0.5304

## Data Availability

All the datasets used in this paper can be found here: https://github.com/dialogtekgeek/AVSD-DSTC10_Official (accessed on 10 August 2021).
